# Fluorogenic protein labeling using a genetically encoded unstrained alkene†
†Electronic supplementary information (ESI) available. See DOI: 10.1039/c6sc03635j



**DOI:** 10.1039/c6sc03635j

**Published:** 2016-09-26

**Authors:** X. Shang, X. Song, C. Faller, R. Lai, H. Li, R. Cerny, W. Niu, J. Guo

**Affiliations:** a Department of Chemistry , University of Nebraska-Lincoln , Lincoln , NE 68588 , USA . Email: jguo4@unl.edu; b Department of Chemical & Biomolecular Engineering , University of Nebraska-Lincoln , Lincoln , NE 68588 , USA . Email: wniu2@unl.edu

## Abstract

We developed a new fluorogenic bioorthogonal reaction that is based on the inverse electron-demand Diels–Alder reaction between styrene (an unstrained alkene) and a simple tetrazine. The reaction forms a new fluorophore with no literature precedent. We have identified an aminoacyl-tRNA synthetase/tRNA pair for the efficient and site-specific incorporation of a styrene-containing amino acid into proteins in response to amber nonsense codon. Fluorogenic labeling of purified proteins and intact proteins in live cells were demonstrated. The fluorogenicity of the styrene–tetrazine reaction can be potentially applied to the study of protein folding and function under physiological conditions with low background fluorescence interference.

## Abstract 2

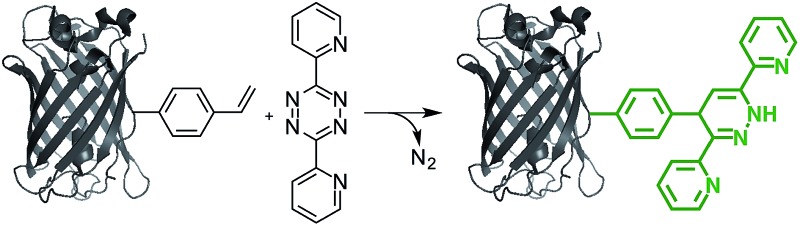
A new fluorogenic bioorthogonal reaction between styrene (an unstrained alkene) and a tetrazine was developed.

## Introduction

Selective labeling of proteins through fluorogenic bioorthogonal reactions is a powerful tool for studying protein structure and function.^1–4^ Fluorogenicity, which leads to good signal-to-noise ratio, is highly desirable for protein labeling in a complex biological environment. Fluorogenic bioorthogonal reactions, where the removal of unreacted reagents is not necessary, could simplify and, in some situations, enable real-time imaging experiments in live cells. One widely used strategy to design fluorogenic bioorthogonal reactions is based on the removal of a specific functional group that suppresses fluorescence of a fluorophore. In this case, the fluorescence quencher is also the reactive group on the reagent, *e.g.*, azide,^5–8^ alkyne,^9^ or tetrazine.^10,11^ This strategy has been applied to fluorogenic protein labeling.^6,8,11^ Another strategy is based on the simultaneous generation of a fluorophore through a bioorthogonal chemical transformation. Due to the challenging aspects in reaction design, this strategy is much less explored. One rare and elegant example is the light-induced 1,3-dipolar cycloaddition reaction between tetrazoles and terminal alkenes, which forms a fluorescent pyrazoline cycloadduct.^12–14^

Here we report a fluorogenic bioorthogonal reaction between styrene and tetrazine. A new fluorophore with no literature precedent is formed in this reaction. In comparison to the fluorescence quencher-removal strategy, which turns a weak fluorescence signal into a stronger one, the fluorophore formation strategy likely gives lower background signal since the bioconjugation product is the only fluorescent species within the entire system. While the styrene–tetrazine reaction is slower than reactions between strained alkenes and tetrazine,^10,11,15–27^ the good cellular stability and the fluorogenic property of the unstrained styrene make it an intriguing alternative to stained alkenes in bioconjugation applications with tetrazines.

## Results and discussion

### The fluorogenic styrene–tetrazine reaction

**Fig. 1:**
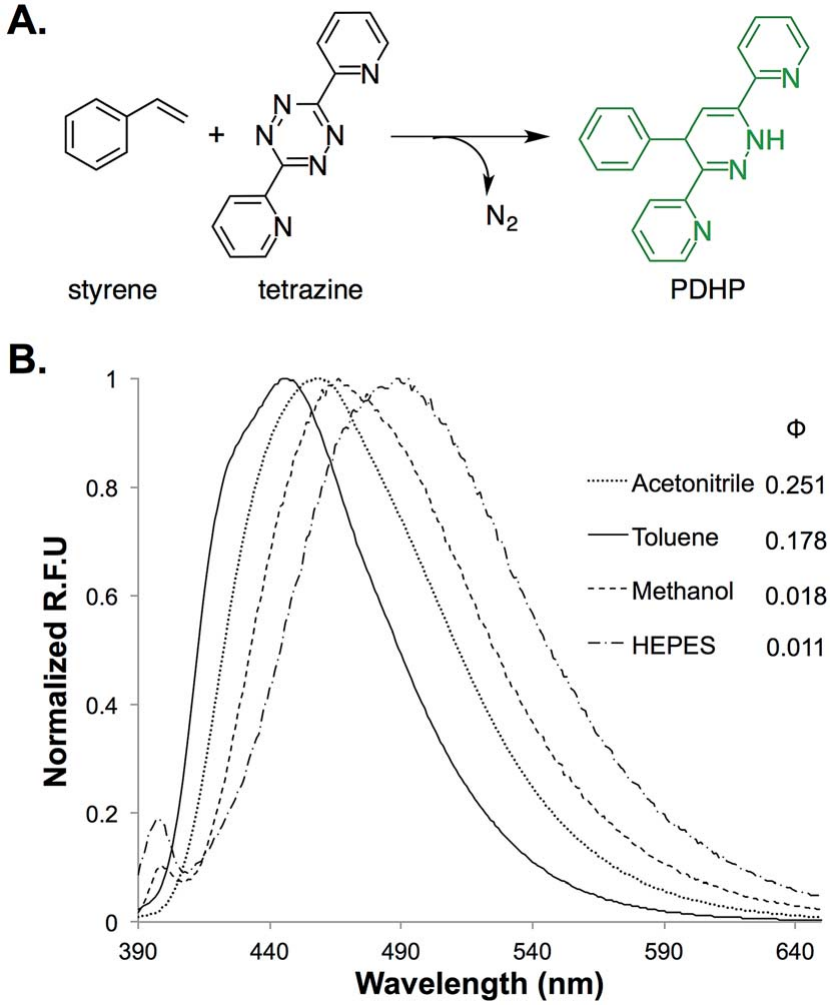
(A) Fluorogenic reaction between styrene and tetrazine; (B) fluorescence properties of the bio-conjugation product, 4-phenyl-3,6-di(pyridin-2-yl)-1,4-dihydropyridazine (PDHP). *λ*_ex_ = 360 nm.

The fluorogenic property of the styrene–tetrazine reaction was discovered during our investigation of inverse electron-demand Diels–Alder (iEDDA) reactions between alkenes and tetrazines. The cycloaddition product of styrene and 3,6-dipyridin-2-yl-1,2,4,5-tetrazine (abbreviated as tetrazine hereafter), 4-phenyl-3,6-di(pyridin-2-yl)-1,4-dihydropyridazine (PDHP), represents a new fluorophore with no literature precedent (Fig. 1). The structure of the molecule was confirmed by both 1D and 2D ^1^H NMR (Fig. S13 and S14†). Our study also showed that PDHP is a solvatochromic fluorophore (Fig. 1B). The absorption spectra and extinction coefficients (3769–4674 M^–1^ cm^–1^) of PDHP are shown in Fig. S2.† The quantum yield of PDHP ranged from 0.011 to 0.251 in solvents of different polarity, which makes PDHP a potential candidate for the study of protein folding and conformational change. Comparing to some commonly used fluorophores^28^ (Table S3†), PDHP has relatively low quantum yield and extinction coefficient. On the other hand, PDHP has a large Stokes shift, which could be beneficial (*e.g.*, less self-quenching and/or autofluorescence background) in certain imaging applications.

^1^H NMR studies showed that PDHP was stable when stored in DMSO/D_2_O (4 : 1) at room temperature for over 24 hours (Fig. S15†). When PDHP was incubated at 37 °C in PBS buffer (pH 7.4, 10% DMSO as cosolvent) in the presence of air, only a slow decay of fluorescence was observed (Fig. S3†). Data of the stability studies (Fig. S3†) also supports that PDHP, not the oxidation product of PDHP (4-phenyl-3,6-di(pyridin-2-yl)pyridazine), is the true fluorophore. To further characterize PDHP, we conducted pH-sensitivity studies. The emission spectra of PDHP were monitored in solutions of varied pH (Fig. S4B†). The fluorescence intensity peaked between pH 7 and pH 10, and decreased significantly when the pH was lower than 5. Notable changes in the absorbance spectrum of PDHP solution were also observed at the lower pH (Fig. S4A†). The fluorescence of PDHP was not affected by common nucleophiles in the biological system, such as cysteine and glutathione (Fig. S5†). This property makes the PDHP-forming styrene–tetrazine reaction compatible with protein labeling in live cells.

The fluorogenic mechanism of the styrene–tetrazine reaction is completely different from previously reported fluorogenic reactions involving tetrazine,^11^ where the fluorescence quenching effect of tetrazine to a covalently linked fluorescent probe was exploited.^29^ The loss of the tetrazine moiety results in the increase of the fluorescence signal from the probe. In comparison to this quencher-removal strategy, which turns a weak fluorescence signal into a stronger one, the *in situ* fluorophore-forming reaction between styrene and tetrazine has the minimal background signal since the conjugation product is the only fluorescent species within the entire system.

### Reaction rate of the styrene–tetrazine reaction

To estimate if styrene–tetrazine reaction can be applied to the labeling of biomolecules in live cells, we conducted kinetics studies of the styrene–tetrazine reaction in methanol/water (v/v 1 : 3). The pseudo-first-order rate constant (*k*_obs_) was measured by monitoring the consumption of tetrazine in the presence of different concentrations of excess styrene. The second-order rate constant was determined by plotting *k*_obs_ against styrene concentrations. The styrene–tetrazine reaction (*k* = 0.078 M^–1^ s^–1^) is faster than reactions between isolated terminal alkenes and tetrazines (entry 1–3, Table S1†).^30–33^ This observation is consistent with results of our quantum mechanical calculations (Table S2†), which showed that a C-substituent with π conjugation (*i.e.*, phenyl-) raised the HOMO energy of a terminal alkene.^34^ HOMO (alkene) of higher energy level benefits an iEDDA reaction between an alkene and a tetrazine.^35,36^ Although the styrene–tetrazine reaction is slower than certain reactions between strained alkenes and tetrazine (entry 7, 8, 10, Table S1†),^10,11,15–27^ its rate is comparable to the strain-promoted cycloaddition of fluorinated cyclooctynes with azides^37^ (entry 11, Table S1†) and the first generation of cyclopropene–tetrazine reaction (entry 9, Table S1†),^26^ which have been successfully applied to the labeling of biomolecules in live cells.^26,30,31,33,37^

### Genetic incorporation of KStyr

**Fig. 2:**
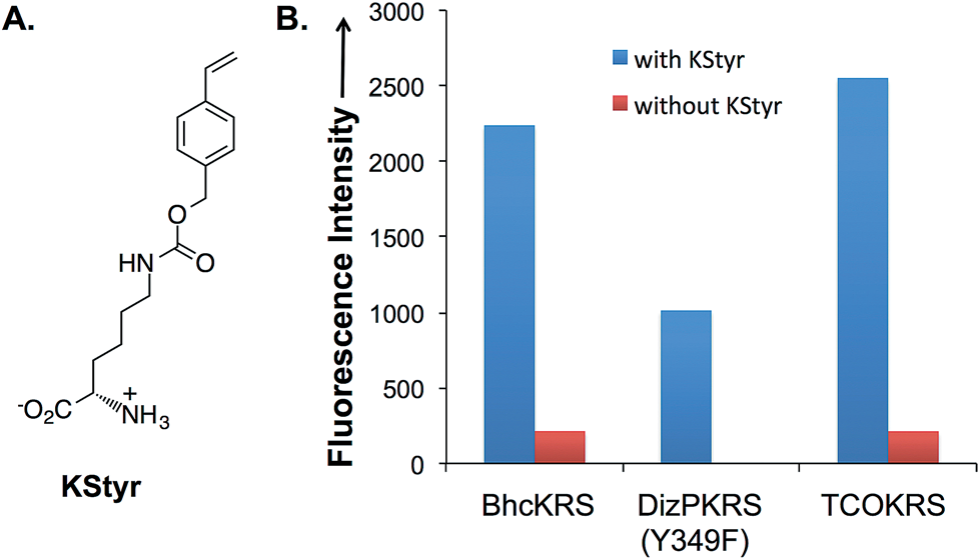
Genetic incorporation of KStyr in *E. coli*. (A) Structure of KStyr (4-vinylbenzyl-*N*-carbamoyl-l-lysine); (B) fluorescence readings of cells expressing PylRS variants and a sfGFP-Asn149TAG mutant. The expressions were conducted either in the presence or in the absence of 0.5 mM KStyr. Fluorescence intensity was normalized to cell growth.

In order to apply the fluorogenic bioorthogonal styrene–tetrazine reaction to protein labeling, a lysine-derived unnatural amino acid containing styrene moiety (KStyr; Fig. 2A) was synthesized. We screened a library of reported pyrrolysyl-tRNA synthetase (PylRS) mutants to identify ones that could aminoacylate an amber suppressor tRNA (tRNA_CUA_) with KStyr in *E. coli*. The amber suppression efficiency was directly linked to the expression level of a GFP mutant (sfGFP-Asn149TAG) that has an amber nonsense codon at position Asn149.^38,39^ Among all the PylRS variants examined (Fig. S7†), three (BhcKRS,^40^ DizPKRS-Y349F,^41^ and TCOKRS^11^) supported the efficient synthesis of full-length sfGFP (Fig. 2B). The DizPKRS-Y349F mutant (L274A, C313S, Y349F)^41^ was chosen for future work. This synthetase displayed the best fidelity towards KStyr and good suppression efficiency. In the absence of KStyr, no sfGFP fluorescence was detected (Fig. 2B). In a large-scale (100 mL cell culture) expression experiment of the sfGFP mutant (sfGFP-N149KStyr) in *E. coli*, 23 mg L^–1^ of the fluorescent protein was obtained after partial purification using affinity chromatography (Fig. S8†). Mass spectrometry analyses of the purified protein (Fig. S9†) confirmed that KStyr was site specifically incorporated at position 149 of sfGFP.

### *In vitro* protein labeling

We first conducted a series of labeling experiments to gauge the reaction between protein-borne styrene group and tetrazine reagent. A previously reported tetrazine–fluorescein reagent^30^ (FL–Tet, Fig. S1†) was synthesized and used in these studies. Following labeling reactions of sfGFP-N149KStyr by FL–Tet, samples were boiled to denature the protein so that the only fluorescent species is the fluorescein conjugates. Protein band with fluorescence was detected 2 min after the reaction was initiated and the fluorescence intensity increased in a time-dependent manner (Fig. S10B†). Control experiments using wild-type sfGFP and FL–Tet, or sfGFP-N149KStyr only did not afford detectable labeling (Fig. S10†). These results demonstrate that the unnatural styrene moiety of KStyr is biocompatible and orthogonal to functional groups in natural amino acids.

**Fig. 3:**
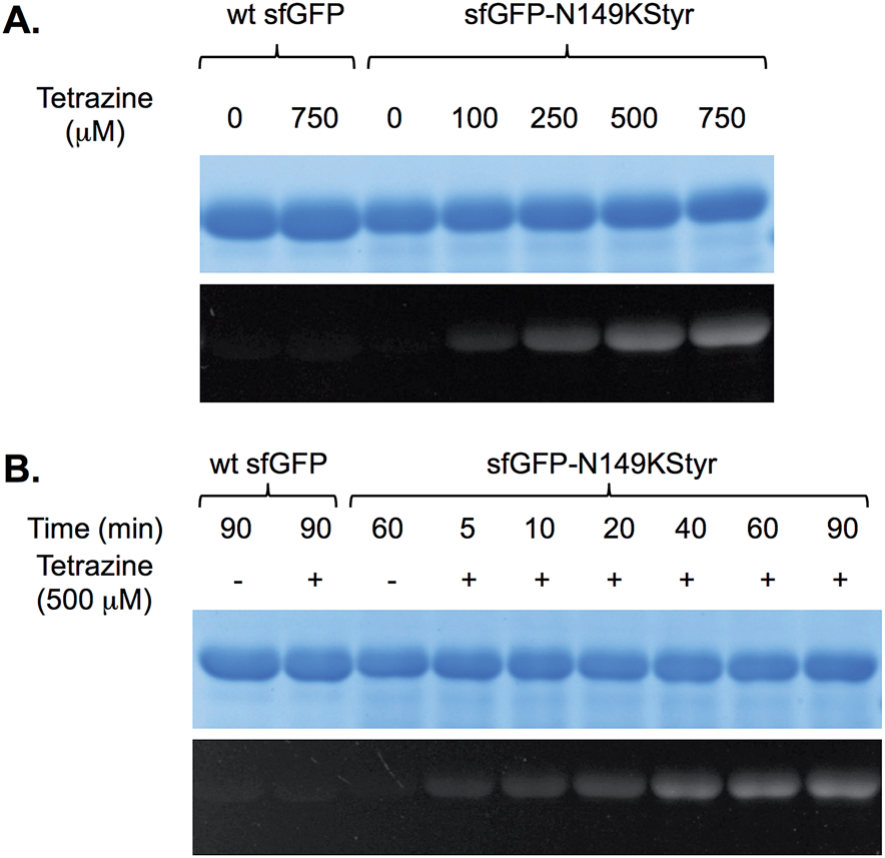
Fluorogenic labeling of sfGFP variants with tetrazine. Following labeling reactions, protein samples were denatured by heating, then analyzed by SDS-PAGE. The top panel in each figure shows Coomassie blue stained gel and the bottom panel shows the fluorescent image of the same gel before Coomassie blue treatment. (A) Labeling of sfGFP-N149KStyr mutant with varied concentrations of tetrazine for 10 minutes. Protein samples (2.75 μg) after labeling reactions were analysed by SDS-PAGE; (B) reaction progress of sfGFP-N149KStyr labeling with 500 μM of tetrazine. Wild-type sfGFP was included in both experiments as the control.

Encouraged by the initial results, we further examined if the PDHP fluorophore generated from the styrene–tetrazine reaction could be directly detected in protein labeling experiments. We first examined the labeling of the sfGFP-N149KStyr mutant with varied concentrations of tetrazine in PBS buffer following a 10 min reaction (Fig. 3A). Weak fluorescence was detected when 100 μM of tetrazine was used. Significantly greater fluorescence intensities were observed as tetrazine concentrations reached 250 μM or higher (Fig. 3A). A robust fluorogenic protein labeling was also observed in a time dependence study using 500 μM of tetrazine. As shown in Fig. 3B, fluorescence was detected 5 min after the reaction was initiated. The fluorescence intensity increased gradually in a time-dependent manner (Fig. 3B and S16†). No fluorescence was observed in control experiments when either wild-type sfGFP was used in the reaction or tetrazine was omitted in reactions involving sfGFP-N149KStyr. Based on mass spectrometry studies, the correct mass of sfGFP-N149KStyr protein after the labelling reaction was observed (calculated mass: 27 673; observed mass: 27 673; these masses are corresponding to protein without N-terminal methionine). Above results confirmed that this fluorogenic styrene–tetrazine reaction could be used as an efficient tool to selectively label a purified protein.

### *In vivo* protein labeling

**Fig. 4:**
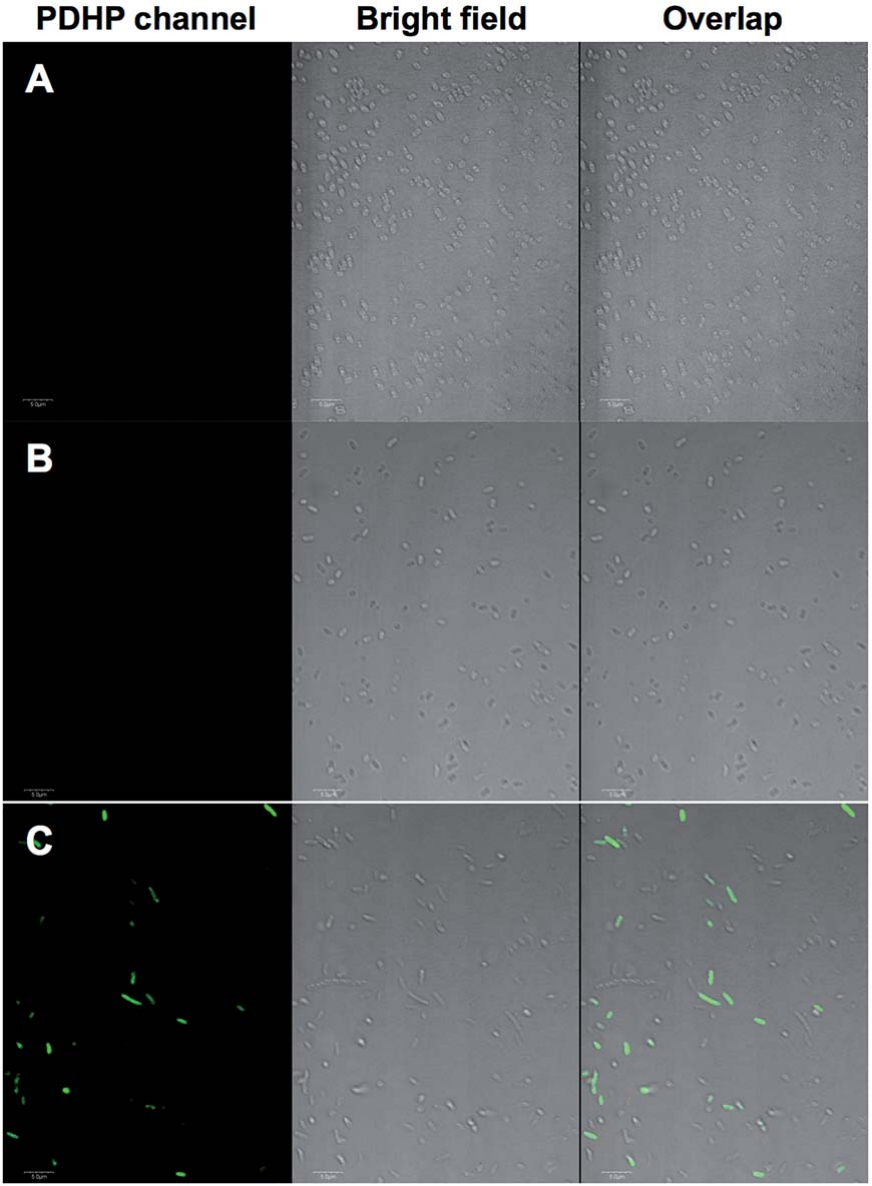
Selective labeling of *E. coli* cells expressing HdeA-F28KStyr. (A) Wild-type HdeA that was expressed in the presence of KStyr; (B) HdeA-F28KStyr mutant that was expressed in the presence of KStyr but in the absence of DizPKRS-Y349F; (C) HdeA-F28KStyr mutant that was expressed in the presence of KStyr and DizPKRS-Y349F. For all images, the left panel shows fluorescent images of *E. coli* cells in PDHP channel (405 nm excitation and 505–540 nm emission), the middle panel shows bright-field images of the same *E. coli* cells, and the right panel shows composite images of bright-field and fluorescent images. Scale bars, 10 μm.

We demonstrated that the fluorogenic styrene–tetrazine reaction could be used to label an intracellular stress response protein, HdeA, in live cells. Plasmid pHdeA was constructed to encode an HdeA mutant containing KStyr at position 28 (HdeA-F28KStyr). *E. coli* cells expressing HdeA-F28KStyr was washed and incubated with 100 μM tetrazine for 1.5 hour at 37 °C. Cells were collected, directly re-suspended PBS buffer (without additional washing steps), and imaged. As shown in Fig. 4C, strong fluorescent signals that co-localized nicely with cells were detected. As a control, *E. coli* cells expressing wild-type HdeA in the presence of KStyr was washed and incubated with 100 μM tetrazine under the same conditions. No fluorescence was observed (Fig. 4A). As a second control, fluorescence was also not detected from cells expressing HdeA-F28KStyr in the presence of KSTyr but in the absence of DizPKRS-Y349F (Fig. 4B). The above two control experiments confirmed that the observed fluorescence signals in Fig. 4C were from labeled HdeA-F28KStyr mutant protein and not from free KStyr. In comparison to labeling reagents that are constantly fluorescent, the fluorogenic styrene–tetrazine reaction does not require an extra washing step to remove excess fluorescent reagents. Furthermore, the preparation of a bioorthogonal reagent–fluorophore conjugate is not needed, which simplifies the labeling of intracellular proteins.

## Conclusions

In conclusion, a novel PDHP fluorophore with intriguing photophysical properties was formed from the styrene–tetrazine reaction. The successful genetic incorporation of a styrene-derived unnatural amino acid (KStyr) enabled site-specific and fluorogenic labeling of proteins both *in vitro* and *in vivo*. While the new PDHP fluorophore and its further application as an solvatochromic dye is still under investigation, the unique fluorogenic property of the styrene–tetrazine reaction could enable protein labeling in live cells without the need of extensive washing steps, which will likely have wide applications in biological studies. Given its ease of preparation, good cellular stability, and unique fluorogenic bioconjugation reaction with tetrazine, styrene serves as an intriguing alternative to strained alkenes for general labeling of biomolecules.
