# Supplementary material for: Fluorogenic protein labeling using a genetically encoded unstrained alkene
Source: Chem Sci. 2016 Sep 26;8(2):1141–5. doi: 10.1039/c6sc03635j (PMC5369545; doi:10.1039/c6sc03635j)
Supplement: Supplementary file 1 [file SC-008-C6SC03635J-s001.pdf]

Support Information

I. General material and method.. 2

II. Experimental procedure...3

III. Supplemental tables... 9

IV. Supplemental figures

1. Figure S1.. 11

2. Figure S2.. 12

3. Figure S3.. 13

4. Figure S4.. 14

5. Figure S5.. 15

6. Figure S6.. 16

7. Figure S7.. 17

8. Figure S8.. 18

9. Figure S9.. 19

10. Figure S10.. 20

11. Figure S11.. 21

12. Figure S12.. 22

13. Figure S13.. 23

14. Figure S14.. 24

15. Figure S15.. 25

16. Figure S16.. 26

IV. Reference...27

I. General material and method

Unless otherwise noted, starting materials, solvents and reagents for chemical synthesis were obtained from commercial suppliers (Acros, Alfa Aesar, Sigma-Aldrich, Chem-impex) and used without further purification. Dry solvents were either freshly distilled by following standard methods or directly purchased from Acros. Deuterated solvents were obtained from Sigma-Aldrich. Flash chromatography (FC) was carried out using SiliaFlash P60 (0.04–0.063 mm, 230–400 mesh) from Silicycle. Thin layer chromatography (TLC) was performed on glass-backed, precoated silica gel plates (Analtech). NMR spectra were recorded at 25 °C using a Bruker Advance III-HD 400 MHz NMR. Chemical shifts were reported in ppm with deuterated solvents as internal standards ($CDCl_3$, H 7.26, C 77.0; DMSO-d_6, H 2.50, C 39.5; D_2O, 4.79). Multiplicity was reported as follows: s = singlet, d = doublet, t = triplet, q = quartet, m = multiplet, b = broad. UV absorbance measurements for kinetic studies were conducted on Shimadzu UV2401-PC. Absorbance spectrum and intensity were measured on Shimadzu UV2401-PC and Thermo Scientific GENESYS 10S UV/Vis Spectrophotometer. Fluorescence spectrum and intensity were recorded on Horiba FluoroMax 4 spectrometer and BioTek Synergy H1 Hybrid plate reader. Sodium dodecyl sulfate-polyacrylamide gel electrophoresis (SDS-PAGE) was performed on Bio-Rad mini-PROTEAN electrophoresis system. Bio-Rad Prestained Protein Ladder was applied to at least one lane of each gel for the estimation of apparent molecular weights. Protein gels were stained by Coomassie Brilliant Blue staining and visualized using Bio-Rad Molecular Imager ChemiDoc XRS+ System. For in-gel fluorescence imaging, either Bio-Rad Molecular Imager ChemiDoc XRS+ System or GE Typhoon FLA9500 was used. Live cells were imaged on Olympus FV500 inverted (Olympus IX-81) confocal microscope.

II. Experimental procedures

Synthetic procedures

N$^{\alpha}$-(9-Fluorenylmethoxycarbonyl)-N$^{\varepsilon}$- (((4-vinylbenzyl)oxy)carbonyl)-L-lysine methyl ester (2):

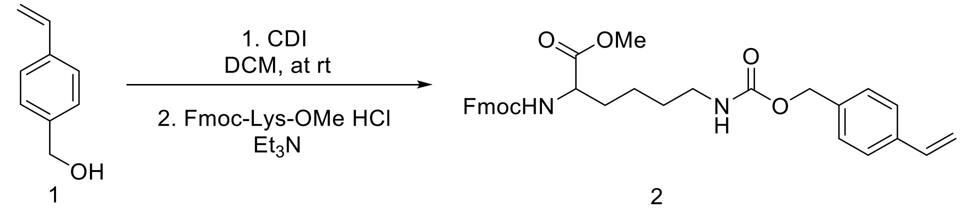

To the solution of 4-vinylbenzyl alcohol (**1**)[1] (268mg, 2.0 mmol) in 5 mL dry dichloromethane, 1,1′-carbonyldiimidazole (CDI; 356mg, 2.2 mmol) dissolved in 5 mL dichloromethane was added with stirring at 0 °C. After the formation of white precipitate, the suspension was kept stirring at room temperature for 1 hour. A solution of N$^{\alpha}$-(fmoc)-L-lysine methyl ester hydrochloric acid salt (838 mg, 2 mmol) in a mixture of triethylamine (Et_3N, 279 µL, 2 mmol) and dichloromethane (DCM, 10 mL) was added to the stirred suspension dropwise. The resulting clear mixture was stirred at room temperature overnight. Following addition of 30 mL dichloromethane, the reaction mixture was washed with 1 N HCl, saturated $NaHCO_3$, and brine sequentially. Resulting solution was dried over Na_2SO_4 and concentrated under vacuum. The residue was further purified by silica gel flash chromatography (dichloromethane/methanol, from 100/1 to 50/1, v/v). Compound **2** (577 mg) was obtained as white solid in 53% yield. ^{1}H-NMR (400 MHz, $CDCl_3$) δ 7.76 (d, *J* = 7.5 Hz, 2H), 7.59 (d, *J* = 7.5 Hz, 2H), 7.28-7.50 (m, 8H), 6.69 (dd, *J* = 10.8, 17.6 Hz, 1H), 5.73 (d, *J* = 17.6 Hz, 1H), 5.36 (br, 1H), 5.25 (d, *J* = 10.8 Hz, 1H), 5.06 (s, 2H), 4.78 (s, 1H), 4.30-4.55 (m, 3H), 4.21 (t, *J* = 6.7 Hz, 1H), 3.75 (s, 3H), 3.19 (m, 2H), 1.30-1.80 (m, 6H). ^{13}C-NMR (400 MHz, $CDCl_3$) δ 173.0, 156.6, 156.1, 144.0, 143.9, 141.4, 137.6, 136.5, 136.2, 128.5, 127.8, 127.2, 126.5, 126.3, 125.2, 120.1, 114.3, 67.1, 66.5, 53.7, 52.6, 47.3, 40.9, 32.3, 29.5, 22.4. HRMS (ESI) calcd for $C_{32}H_{34}N_2O_6$, $[M + H]^+$ 543.2490, found 543.2489.

N$^{\varepsilon}$- (((4-vinylbenzyl)oxy)carbonyl)-L-lysine (KStyr):

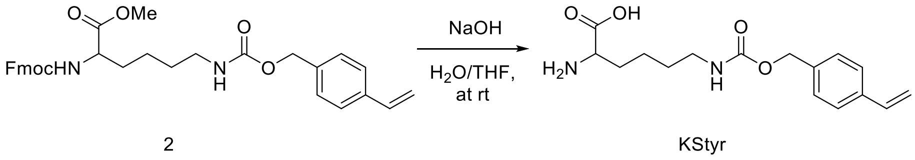

N^{α}-Boc-N^{ε}-(4-vinylphenyl)carbamoyl-)L-lysine methyl ester hydrochloric acid (**2**) (69 mg, 0.13 mmol) was dissolved in tetrahydrofuran (1.3 mL). Sodium hydroxide aqueous solution (0.64 mL, 1 N) was added dropwise with stirring. The mixture was stirred overnight at room temperature, then diluted with water and washed with diethyl ether for five times. The pH of the aqueous layer was adjusted to 7 to afford white precipitate. Vacuum filtration afforded 28 mg KStyr in 72% yield. ^{1}H-NMR (400MHz, 0.5N NaOH/D_2O) δ 7.53 (d, *J* = 8.0 Hz, 2H), 7.40 (d, *J* = 8.0 Hz, 2H), 6.81 (dd, *J* = 11.2, 17.6 Hz, 1H), 5.76 (d, *J* = 17.6 Hz, 1H), 5.35 (d, *J* = 11.2 Hz, 1H), 5.12 (s, 2H), 3.00-3.15 (m, 3H), 1.30- 1.70 (m, 6H). ^{13}C-NMR (100 MHz, D_2O/H_2O/0.2N NaOH) δ: 183.7, 168.4, 158.7, , 137.4, 136.3, 128.1 , 126.5 , 114.7, 66.5, 56.0, 40.4, 34.4, 28.9, 22.3. HRMS (ESI) calcd for $C_{16}H_{22}N_2O_4$, $[M + H]^+$ 307.1653, found 307.1647.

4-phenyl-3,6-di(pyridin-2-yl)-1,4-dihydropyridazine (PDHP)

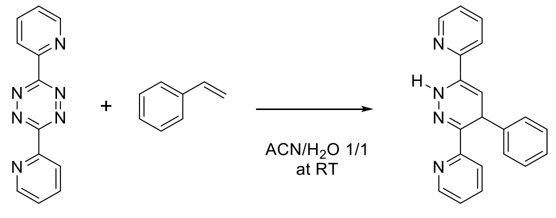

The solution of 3,6-di-2-pyridinyl-1,2,4,5-tetrazine[2] (Tet, 94mg, 0.4 mmol) in 100 mL of acetonitrile (CH_3CN) in water (50:50) was bubbled with Argon under stirring for 30 minutes. Five equivalents of styrene (229 μL, 2 mmol) were added into the solution. The resulting pink mixture was stirred overnight to form yellow solid, which was collected by vacuum filtration as desired product. To improve the final yield, the filtrate was extracted by diethyl ether (3 × 50 mL). Combined organic layers were dried over Na_2SO_4. The solvent was removed by vacuum evaporation. The residue was further purified by flash chromatography with ethyl acetate/hexane (10% to 25%) to afford a yellow solid. PDHP (111 mg) was obtained in 89% yield by combining two portions of yellow solid. ^{1}H-NMR (400 MHz, $CDCl_3$) δ 9.34 (s, 1H), 8.55 (dd, *J* = 4.7, 14.3 Hz, 2H), 8.09 (d, *J* = 8.0 Hz, 1H), 7.67-7.69 (m, 2H), 7.62 (t, *J* = 9.1 Hz, 1H), 7.41 (d, *J* = 7.9 Hz, 2H), 7.21-7.27 (m, 3H), 7.14 (t, *J* = 6.3 Hz, 2H), 5.80-5.83 (m, 1H), 5.56 (d, *J* = 6.2 Hz, 1H). ^{13}C-NMR (100 MHz, $CDCl_3$) δ 155.1, 150.4, 148.4, 148.3, 144.0, 142.0, 136.5, 136.1, 135.9, 128.5, 128.0, 126.5, 123.0, 122.6, 121.0, 118.9, 99.0, 36.8. HRMS (ESI) calcd for $C_{20}H_{17}N_4$, $[M + H]^+$ 313.1448, found 313.1442

Quantum yield determination

Quantum yields (ϕ) of PDHP in different solvents were determined by following a reported procedure.[3] Quinine sulfate in 0.1 M H_2SO_4 was used as the standard to calculate ϕ value.[4]

Kinetic measurements.

The reactions were carried out in indicated solvent under pseudo first-order conditions with 10- to 50-fold excess of styrene and 0.1 mM tetrazine. Tetrazine stock solution (0.2 mM) in 20% methanol aqueous solution and 10 mM styrene stock in 30% methanol aqueous solution were prepared and the two stock solutions were mixed freshly into quartz cuvettes before UV measurement. The consumption of tetrazine was recorded by measuring tetrazine absorbance (A) at 530 nm at 21°C in 30 minutes. The pseudo first-order rate constant k_{obs} (s^{-1}) is the slope of a linear plot of the natural log of absorbance, In(absorbance), against time (t). Triplet independent measurements were carried out for k_{obs} measurements. The second-order rate constant k_2 ($M^{-1}s^{-1}$) is the slope of calibration curves generated by plotting k_{obs} (s^{-1}) against styrene concentration (M^{-1}).

Computational methodology

All the quantum mechanical (QM)/FixSol[5] calculations were performed by using the Quantum chemistry Polarizable force field program (QuanPol)[6] implemented in the General Atomic and Molecular Electronic Structure System (GAMESS).[7] The geometry optimizations were performed by using QM/FixSol[1] methods with density functional theory (DFT). In the calculations, B3LYP (Becke, three-parameter, Lee-Yang-Parr) exchange-correlation functional[8] and the 6-31++G(d,p)[8] basis set were used. The solvent effect was described with the FixSol model with a dielectric constant of 78.39.

Plasmid construction.

pHdeA-F28TAG. HdeA gene was PCR amplified from *E. coli* genomic DNA. An amber mutation (F28TAG) was introduced by site-directed mutagenesis. The HdeA-F28TAG gene was digested with *Nde*I and *Blp*I, and ligated into pLei-GFP[9] vector, which was treated with the

same restriction enzymes, to afford pHdeA-F28TAG. Plasmid pHdeA-F28TAG was confirmed by DNA sequencing.

psfGFP-N149TAG. The sfGFP-N149TAG gene was assembled by overlapping PCR using pLei-sfGFP-Y66TAG plasmid[10] as the template. The digested PCR product was inserted into pLei[9] vector behind a T5 promoter to afford plasmid psfGFP-N149TAG. Following were primers used in the construction:

P1: 5'-GAGGAGAAATTACATATGTCCAAG-3'

P2: 5'- AGTGAGGGTAGTTACCAGGGT-3'

P3: 5'-ACCCTGGTAACTACCCTCACTtatGGTGTCCAGTGCTTCTCTCG-3'

P4: 5'-GAGTCCAAGCTCAGCGGTG-3'

P5: 5'-GTGGCTATTGAAGTTATACTCCA-3'

P6: 5'-TGGAGTATAACTTCAATAGCCACtagGTGTACATCACTGCTGATAAACAG-3'

psfGFP-wt. The plasmid was constructed by site-directed mutagenesis using psfGFP-N149TAG as the template. Following primers were used.

P7 5'-TGGAGTATAACttcAATAGCCACaatGTGTACATCACTGCTGATAAACAG-3'

P8 5'-CTGTTTATCAGCAGTGATGTACACattGTGGCTATTGAAGTTATACTCCA-3'

Screening of pyrrolysyl-tRNA synthetase variants for the genetic incorporation of KStyr.

A plasmid encoding a pyrrolysyl-tRNA synthetase (PylRS) mutant of interest was co-transformed with pLei-sfGFP-N149TAG into *E. coli* GeneHog.[9] The resulting strain was inoculated into 1 mL of LB media with kanamycin (Kan, 50 mg/L) and chloramphenicol (Cm, 34 mg/L). Cells were cultured at 37 ^{o}C with shaking overnight. The seed culture (80 μL) was used to inoculate four cultures in fresh LB media (0.8 mL) containing Kan (50 mg/L), Cm (34 mg/L), and IPTG (0.25 mM). Two cultures also contained KStyr (1 mM). Following cultivation at 37 ^{o}C with shaking for 24 hours, cells were collected by centrifugation, washed with PBS buffer. and resuspended in 0.8 mL PBS for fluorescence and OD_{600} measurements using a Synergy H1 Hybrid plate reader. The fluorescence of sfGFP was monitored with λ_{Ex} = 480 nm and λ_{Em} =

510 nm. The cell density was estimated by measuring the sample absorbance at 600 nm. Fluorescence intensities were normalized to cell growth. A total of 19 pyrrolysyl-tRNA synthetase mutants were characterized.

Protein expression and purification.

E. coli GeneHog strain harboring plasmid pBK-mutant 10 and psfGFP-N149TAG was cultured in 100 mL LB media containing Kan (50 mg/L) and Cm (34 mg/L) at 37 °C with shaking. The protein expression was induced at OD_{600} of 0.6 by the additions of IPTG (0.25 mM) and KStyr (0.5 mM). Following an additional 16 h of cultivation, cells were collected by centrifugation at 5,000g and 4 °C for 15 min. Harvested cells were resuspended in lysis buffer containing potassium phosphate (20 mM, pH 7.4), NaCl (150 mM), and imidazole (10 mM). Cells were subsequently disrupted by sonication. Cellular debris was removed by centrifugation (21,000g, 30 min, 4 °C). The cell-free lysate was applied to Ni Sepharose 6 Fast Flow resin (GE Healthcare). Protein purification followed manufacturer's instructions. Protein concentrations were determined by Bradford assay (Bio-Rad). Purified protein was desalted prior to MS analysis.

Protein mass spectrometry

For full-length protein, the samples were directly analyzed by mass spectrometer. For protein fragment, gel band containing sfGFP-N149KStyr was cut from SDS-PAGE gel stained by Coomassie blue. After in-gel digestion with trypsin, the protein sample was dried down and re-dissolved in 120 μL of aqueous solution with 2.5% acetonitrile and 0.1% formic acid. A 5 μL of the digest sample was injected into a nano-LC-MS/MS that was equipped with a 0.075 mm x 250 mm C18 Dionex column and a Q-Exactive HF mass spectrometer.

The mass spectrometry data was analyzed using Mascot (Matrix Science, London, UK; version 2.5.1). Mascot was set up to search KStyr-containing peptide, LEYNFNSH-KStyr-VYITADK. Deamidation of asparagine and glutamine, oxidation of methionine were specified in Mascot as variable modifications. The MS/MS spectra were searched by Mascot using a fragment ion mass tolerance of 0.060 Da and a parent ion tolerance of 10.0 PPM (Figure S9). The Mascot results were loaded into Scaffold (version Scaffold_4.4.8, Proteome Software Inc., Portland, OR) to validate the MS/MS-based peptide and protein identifications.[11]

Labeling of purified proteins.

sfGFP-N149KStyr (9 μL, 1 mg/mL) in PBS buffer was treated with 1 μL of FL-Tet or Tet (in DMSO) at indicated concentrations. The reaction mixture was incubated at 37 ^{o}C with agitation for indicated period of time. Solution of 5-norbonene-2-methanol (10 μL, 100 mM) in DMSO/PBS (1:9) was added to quench the reaction. The quenched reaction was then mixed with SDS-PAGE sample loading buffer (2x) and heated at 95 ^{o}C for 15 minutes. SDS-PAGE analysis followed manufacture's instruction. Fluorescence detection was performed before staining by Coomassie blue. Protein gels of Tet-labeled protein samples were imaged using Bio-Rad Molecular Imager ChemiDoc XRS+. Protein gels of FL-Tet-labeled protein samples were imaged using GE Typhoon FLA9500. Coomassie blue-stained gels were imaged using Bio-Rad Molecular Imager. As a control, wild-type sfGFP (9 μL, 1 mg/mL) was subjected to labeling and analysis under the same conditions.

Labeling and imaging of live cells.

E. coli cells expressing either wild-type HdeA or HdeA-KStyr mutant proteins were harvested by centrifugation (21,000 g, 5 min, 4 °C). Collected cells were washed three times in the same volume of PBS by vortexing for 5 min. Washed cells were re-suspended in PBS buffer containing 5% glycerol. Tet stock solution was added into cell suspension to a final concentration of 100 μM. After incubation for 90 min at 37 °C with agitation, cells were collected by centrifugation (21,000 g, 5 min, 4 °C) and resuspended in PBS buffer. Cell suspensions were placed on the surface of a glass slide and covered with a glass cover slip for imaging. Cells were imaged on an Olympus FV500 inverted (Olympus IX-81) confocal microscope. PDHP channel was excited by DAPI excitation wavelength (405 nm) and imaged using GFP emission filter (510 nm).

III. Supplemental tables

Table S1. Reaction rate comparison.

entry	reaction	Second-order rate constant *k* (M^{-1} s^{-1})
1	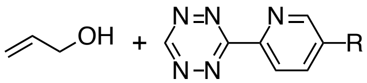	0.0094 (in PBS buffer)[12]
2	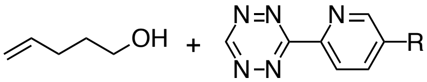	0.036 (in PBS buffer)[12]
3	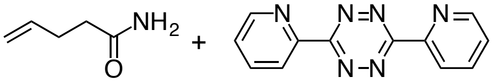	0.0042 (in 1:1 methanol/water)[13]
4	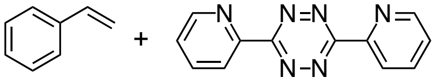	0.048 (in 1:1 methanol/water)[13]
5	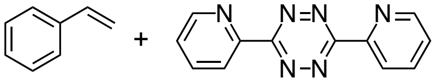	0.078 (in 1:3 methanol/water; this work)
6	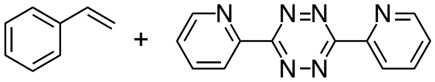	0.131 (in 95:5 water/t-BuOH)[14]
7	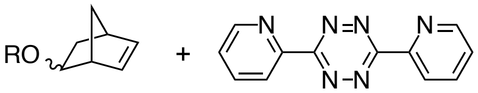	1.0 (in 5:95 methanol/water)[15]
8	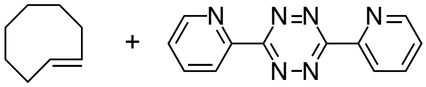	2000 (in 9:1 methanol/water)[16]
9	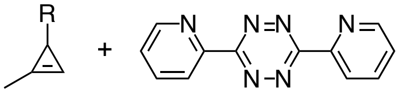	0.05 (in 1:1 CH_3CN/PBS)[17]
10	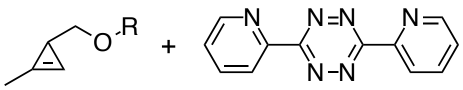	2.8 (in 15:85 DMSO/PBS)[18]
11	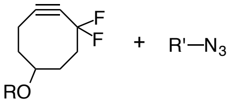	0.076 (in CH_3CN)[19]

Note: These rate constants were measured in different solvents using different methods. The comparison is not absolute.

Table S2. Quantum mechanical calculations of HOMO energies of alkenes.

molecules	HOMO energy (Hartree)
5-hexenol	-0.2526
styrene	-0.2276

Table S3. The photophysical properties of some common fluorophores.[20]

fluorophore	solvent	λ_{ex} (nm)	λ_{em} (nm)	Stokes shift (nm)	ε ($M^{-1}cm^{-1}$)	ϕ
PDHP	MeOH	360	465	105	4329	0.018
PDHP	CH_3CN	360	455	95	3769	0.251
7-Amino-4-methylcoumarin	MeOH	351	430	79	18000	0.75
NBD	MeOH	465	535	70	22000	0.3
fluorescein	pH 9	490	514	24	93000	0.95

IV. Supplemental figures

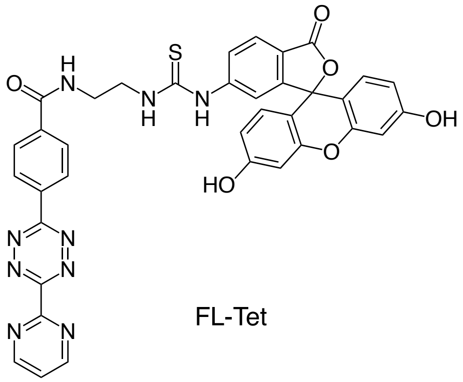

FL-Tet

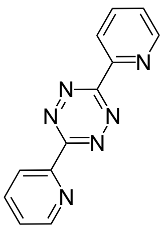

Tetrazine (Tet)

Figure S1. Structures of labeling reagents FL-Tet and tetrazine (Tet). Both compounds were synthesized by following reported procedures. Structures of synthesized compounds were confirmed by ^{1}H NMR.

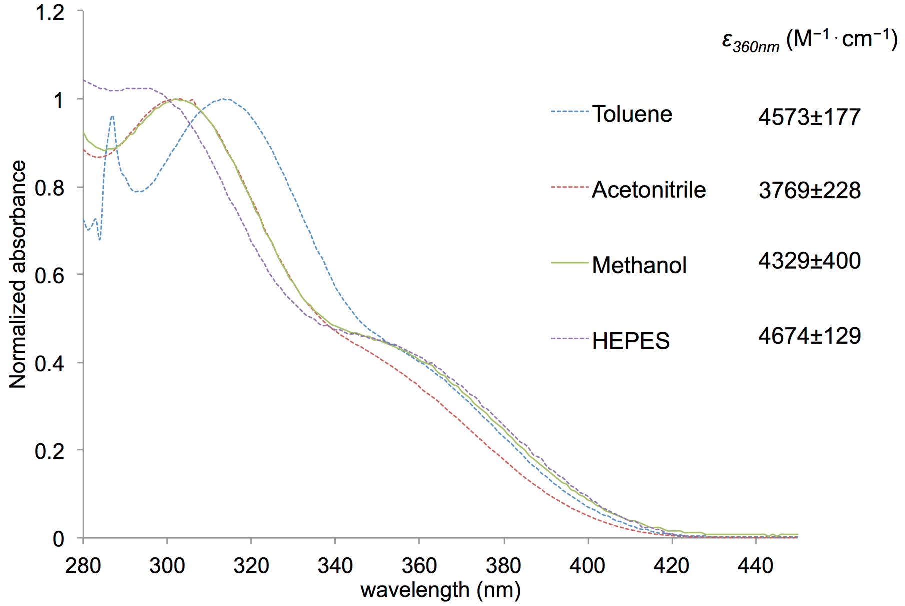

Figure S2. Absorbance spectra and extinction coefficients of PDHP in different solvents. HEPES: 4-(2-hydroxyethyl)-1-piperazineethanesulfonic acid; HEPES buffer contains 5% DMSO as cosolvent to improve the solubility of PDHP.

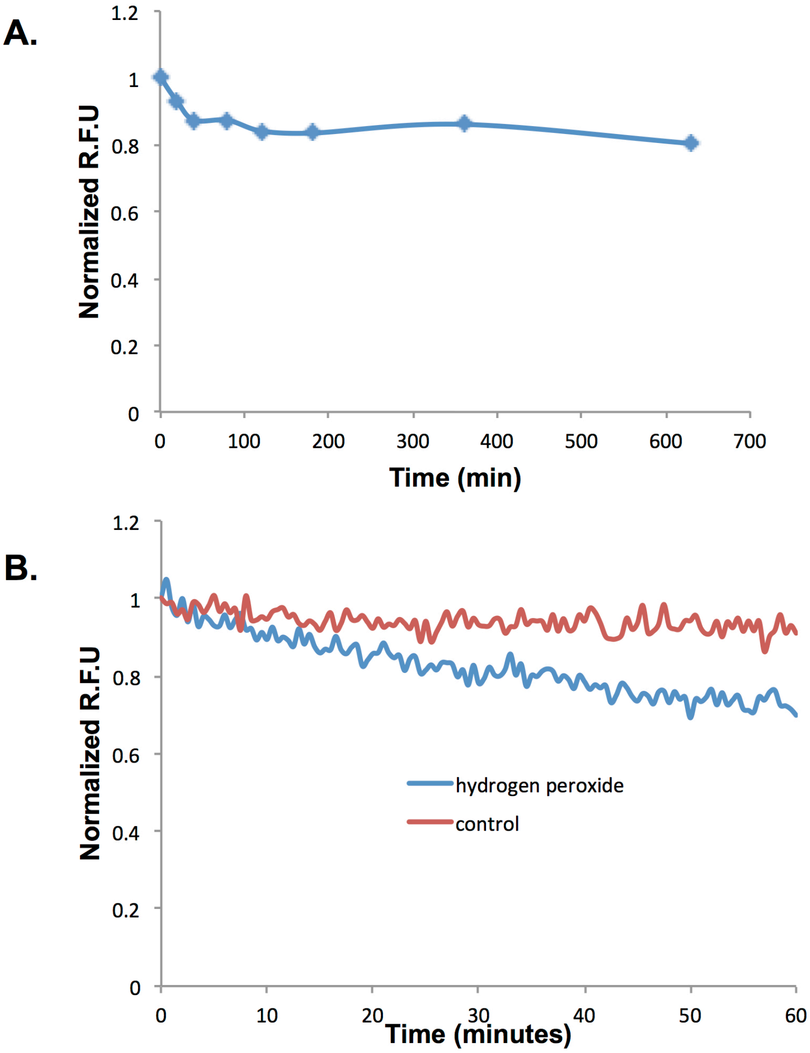

Figure S3. Fluorescence decay of PDHP in PBS buffer in the presence of air or H_2O_2. (A) Fluorescence decay of PDHP with shaking at 37 °C in the presence of atmospheric air. 5 mL of 10 μM PDHP solution in PBS buffer (with 10% DMSO) was incubated at 37 °C with shaking (250 rpm). At an indicated time point, a 100 μL solution was mixed with 100 μL of acetonitrile, and the fluorescence intensity was measured (λ_{Ex} = 360 nm and λ_{Em} = 490 nm.) using a BioTek Synergy H1 Hybrid plate reader; (B) Fluorescence decay of PDHP stored in cuvette at room temperature (21 °C) in the presence of H_2O_2. After addition of H_2O_2 (2 μL, 200 mM), the PDHP solution (2 mL, 10 μM) in PBS buffer (with 10% DMSO) was excited at 360 nm and the intensity of emitted fluorescence light at 490 nm was recorded using a Horiba FluoroMax 4 spectrometer every 30 seconds.

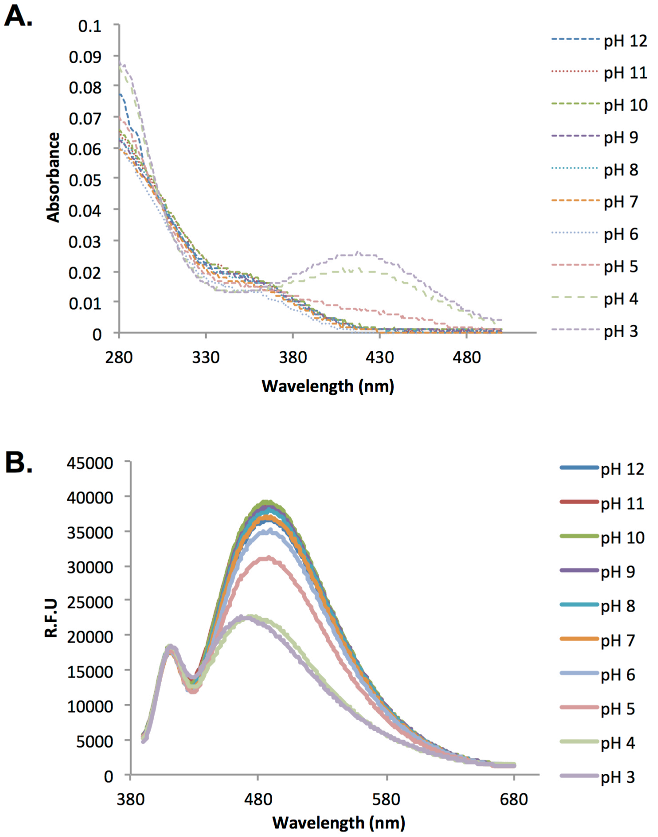

Figure S4. PDHP absorbance and emission spectrum at different pH. Buffers of different pH were prepared by mixing 0.04 M Britton-Robinson buffer (0.04 M acetic acid, 0.04 M phosphoric acid, and 0.04 M boric acid) with 0.2 M NaOH solution at appropriate ratios. All buffered aqueous solutions of PDHP (5 μM) contain 10% DMSO as the cosolvent.

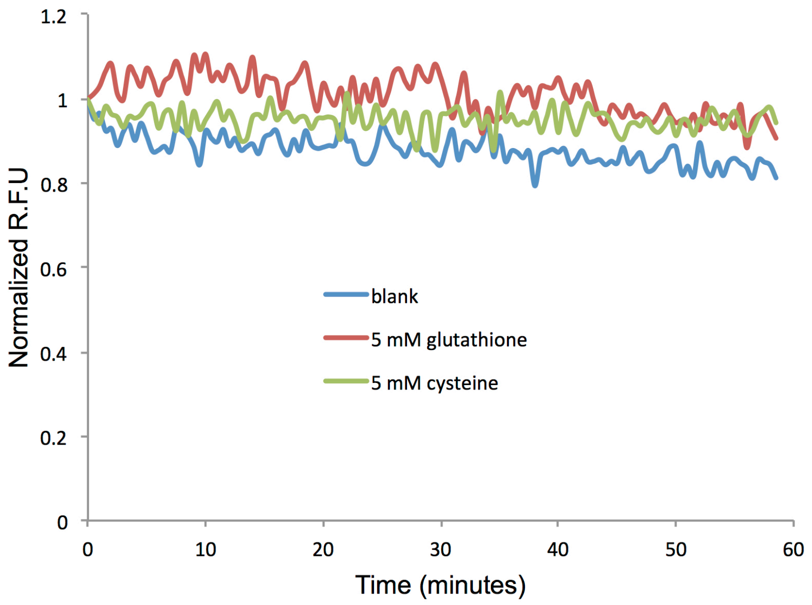

Figure S5. PDHP fluorescence in the presence of cysteine and glutathione. PDHP (20 μM) was incubated with each reagent (5 mM) in PBS buffer (pH 7.4, 20% MeOH) at 21°C. Fluorescence was recorded every 30 seconds using a Horiba Fluoromax 4 spectrometer. Control experiment was carried out without the addition of reagents.

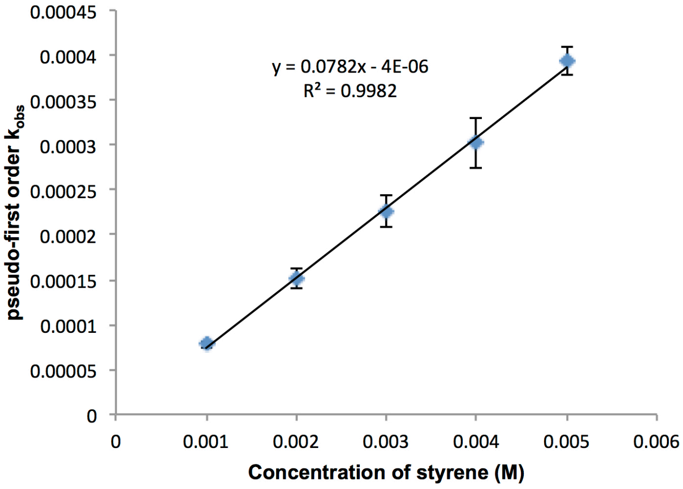

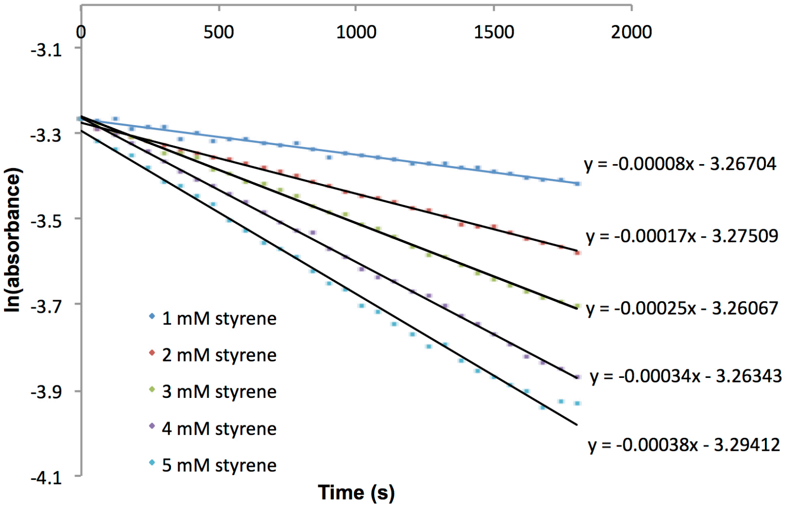

Figure S6. Kinetic studies of styrene-tetrazine reaction in MeOH/H_2O (1:3) solution. (A) Plots of ln(absorbance) of tetrazine against time in reactions with varied concentrations of excess styrene. The slope of the linear curve is the pseudo-first-order rate constant k_{obs} (s^{-1}). The graph represents one dataset of three replicates; (B) Plot of the pseudo-first-order rate constant k_{obs} (s^{-1}) against the concentration of styrene. The slope of the linear curve is the second-order rate constant ($M^{-1}s^{-1}$) of this reaction. The error bar represents the standard deviation from triplicate measurements.

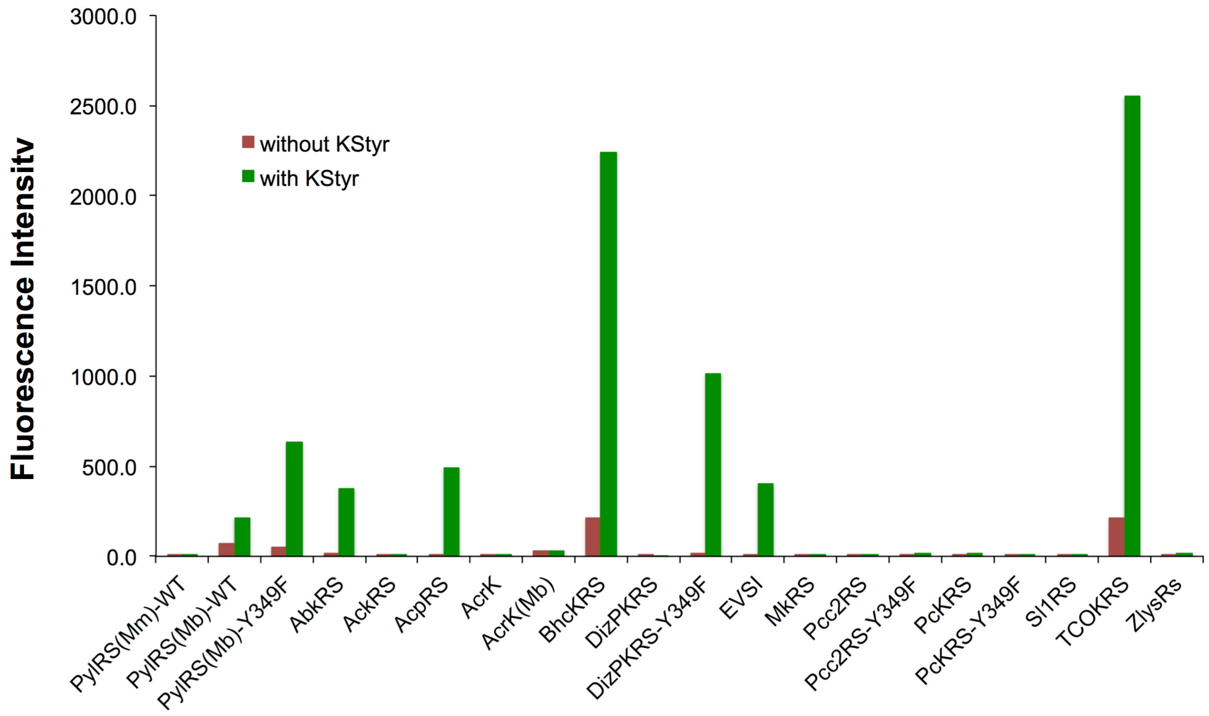

Figure S7. Screening of PylRS mutants using GFP fluorescence assays. Fluorescence intensity was normalized to cell growth.

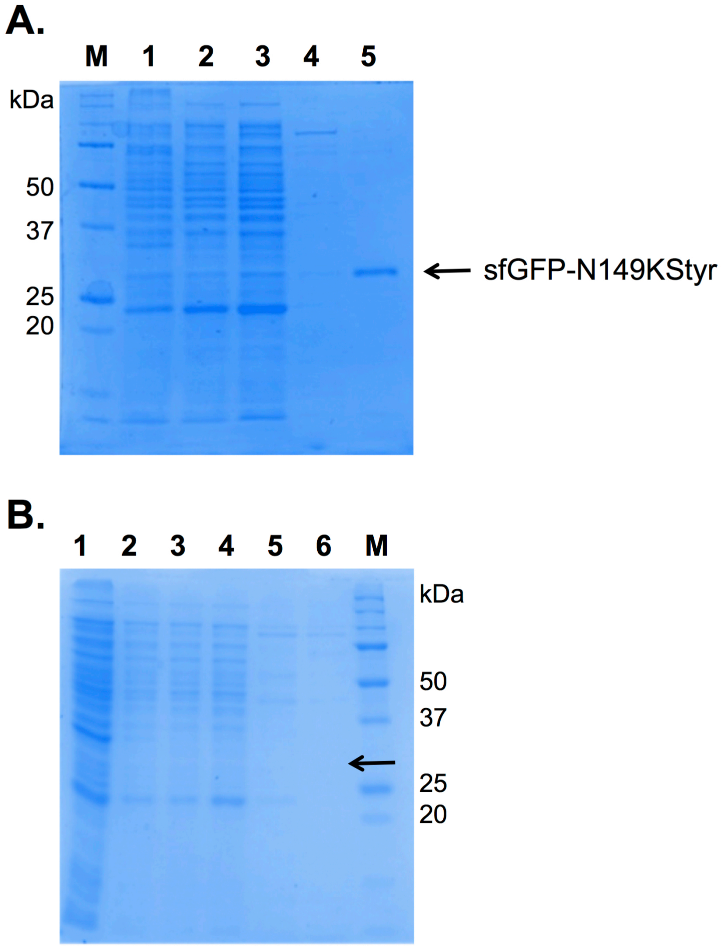

Figure S8. Protein purification and SDS-PAGE analysis. (A) Protein expression (in the presence of 0.5 mM KStyr), purification (through affinity chromatography using Ni-NTA resin), and SDS-PAGE analysis of sfGFP-N149TAG. Lane 1, total soluble proteins after sonication; 2 flow-through fraction; 3, first wash fraction; 4, second wash fraction; 5, elution. The purified sfGFP-N149KStyr is indicated with an arrow (27.6 kDa), and the protein yield is 23 mg/L; (B) Protein expression (in the absence of KStyr), purification (through affinity chromatography using Ni-NTA resin), and SDS-PAGE analysis of sfGFP-N149TAG. Lane 1, total soluble proteins after sonication; 2 flow-through fraction; 3, first wash fraction; 4, second wash fraction; 5, first elution; 6. Second elution. No full-length sfGFP mutant protein was observed.

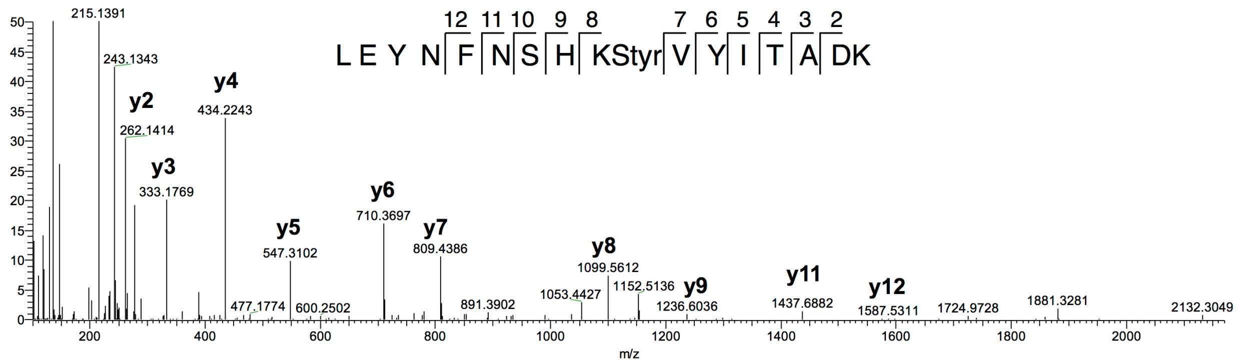

Figure S9. Mass spectrometry analysis of sfGFP-N149KStyr. The y ions are marked in the spectrum. The amino acid sequence of the peptide fragment, LEYNFNSH-KStyr-VYITADK, from mutant sfGFP containing KStyr is shown on top.

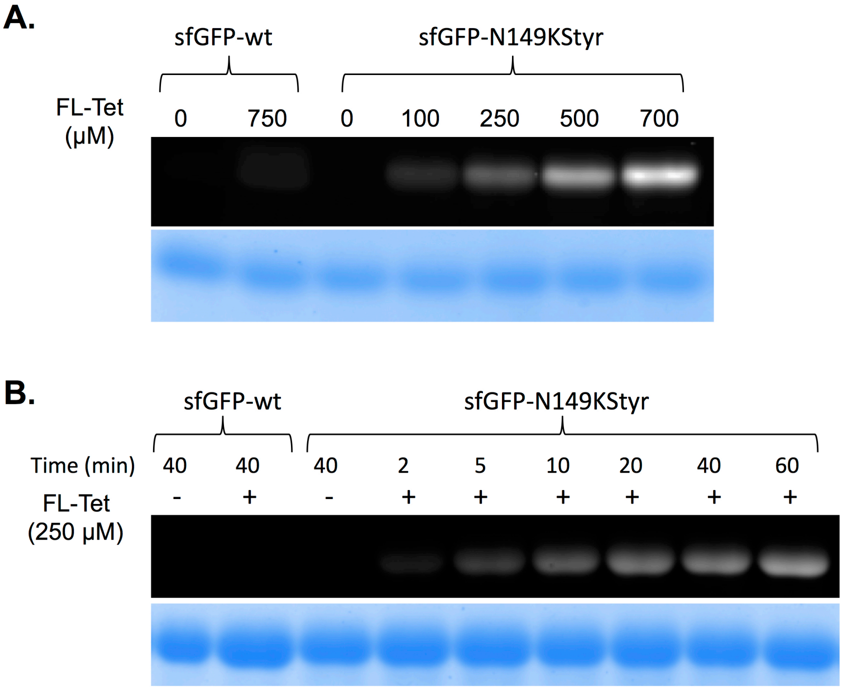

Figure S10. Labeling of sfGFP variants with FL-Tet. Wild-type sfGFP was included in both experiments as the control. Following labeling reactions, protein samples were denatured by heating, then analyzed by SDS-PAGE. The bottom panel in each figure shows Coomassie blue stained gel and the top panel shows the fluorescent image of the same gel (by GE Typhoon imager; Excitation/Emission filter: LPB (510LP)) before Coomassie blue treatment. (A) Labeling of sfGFP-N149KStyr with varied concentrations of FL-Tet for 5 minutes; (B) Reaction progress of sfGFP-N149KStyr labeling with 250 μM of FL-Tet.

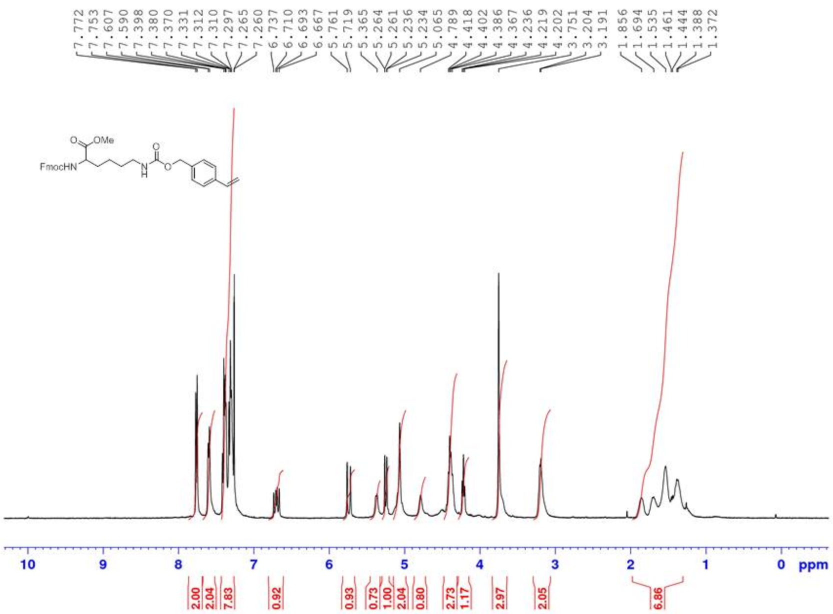

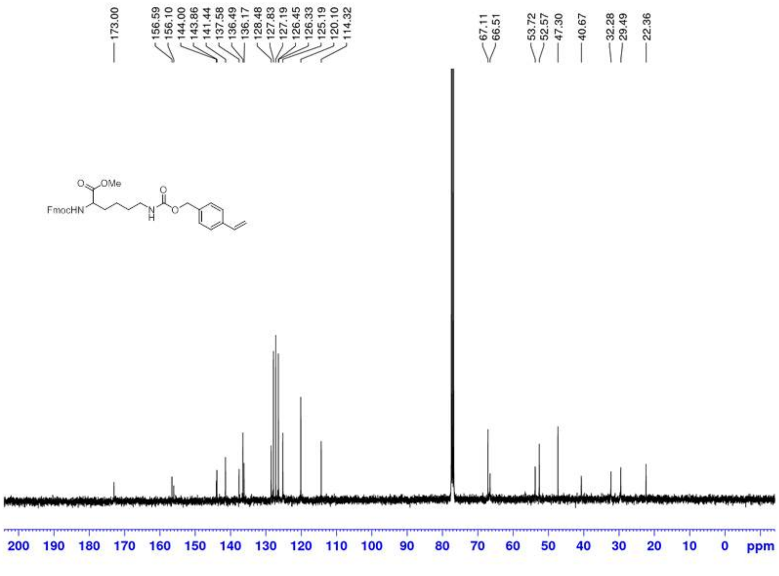

Figure S11. ^{1}H and ^{13}C NMR spectra of protected KStyr.

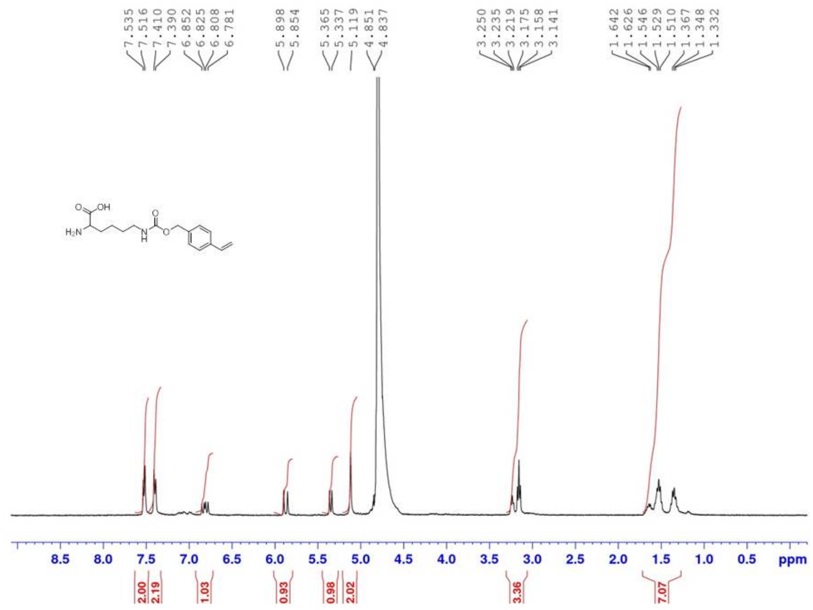

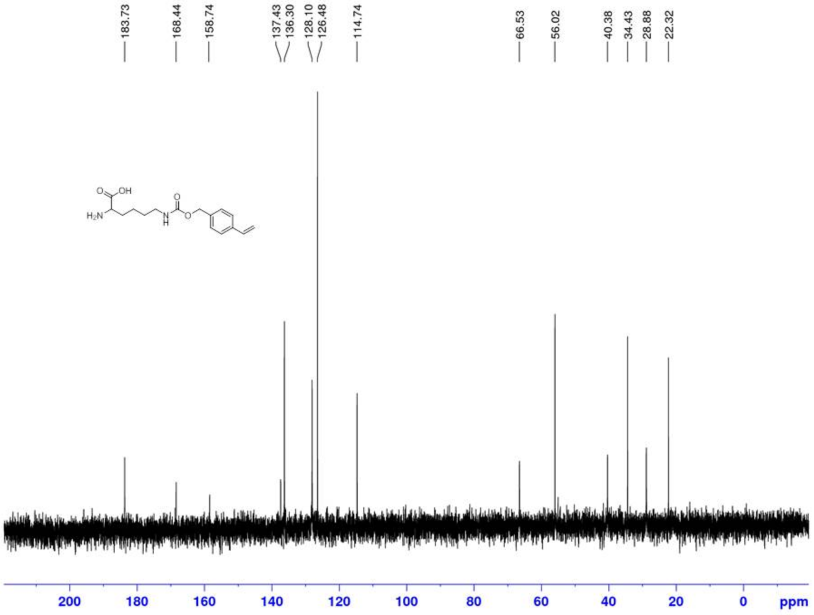

Figure S12. ^{1}H and ^{13}C NMR spectra of KStyr.

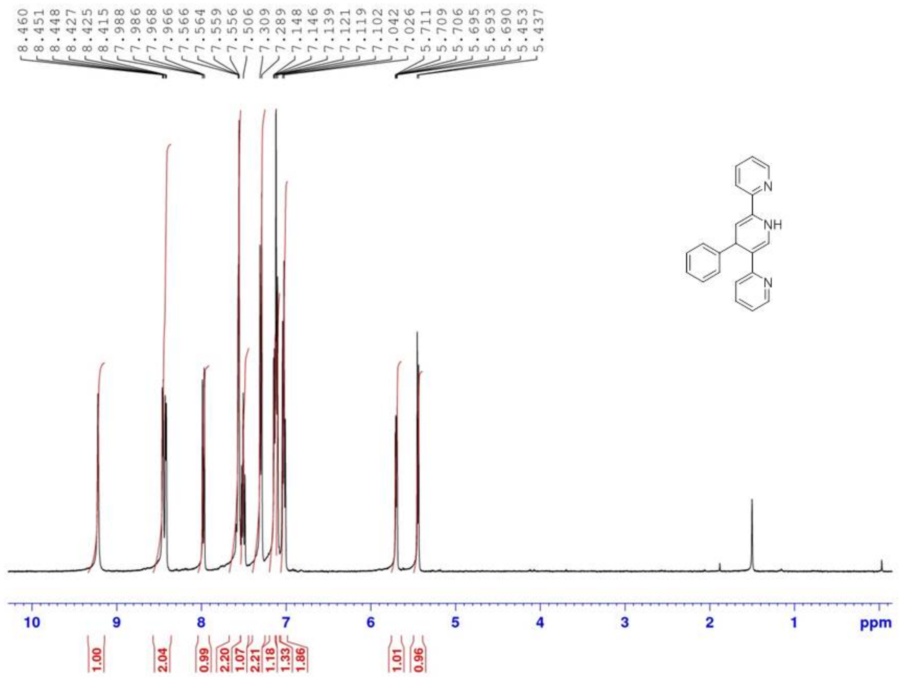

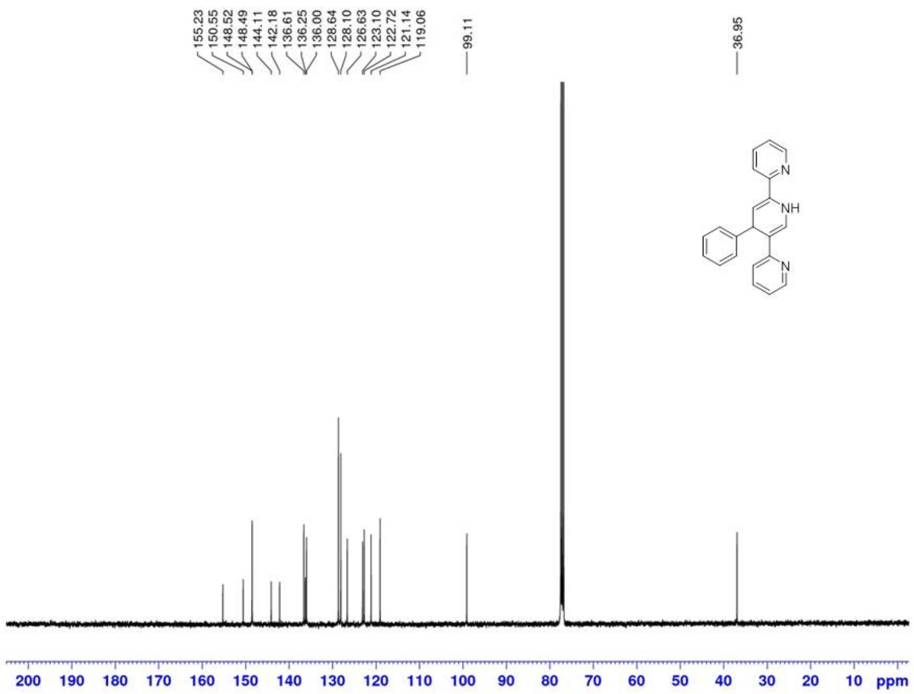

Figure S13. **^{1}H and ^{13}C NMR spectra of 4-phenyl-3,6-di(pyridin-2-yl)-1,4-dihydropyridazine (PDHP).**

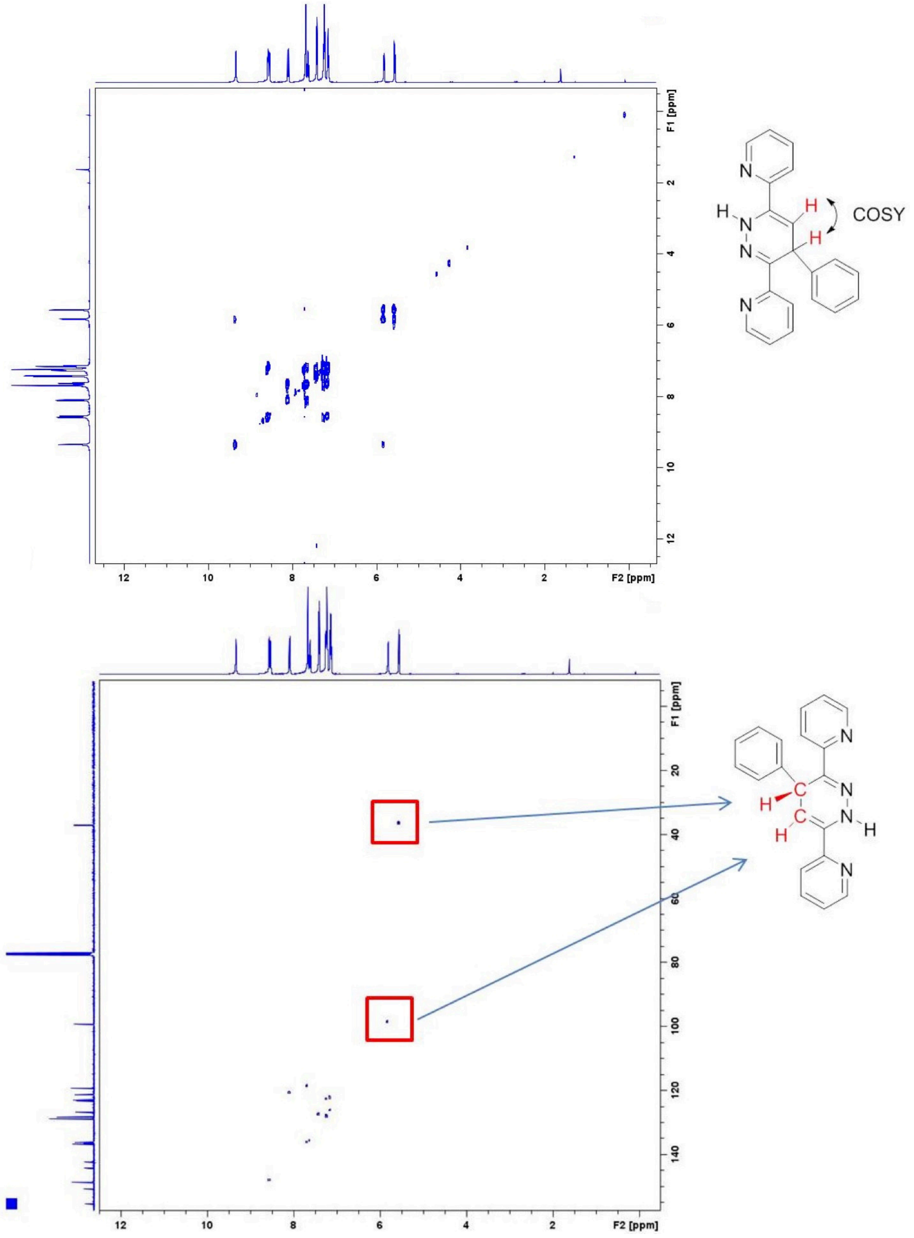

Figure S14. COSY and HSQC analyses of 4-phenyl-3,6-di(pyridin-2-yl)-1,4-dihydropyridazine (PDHP).

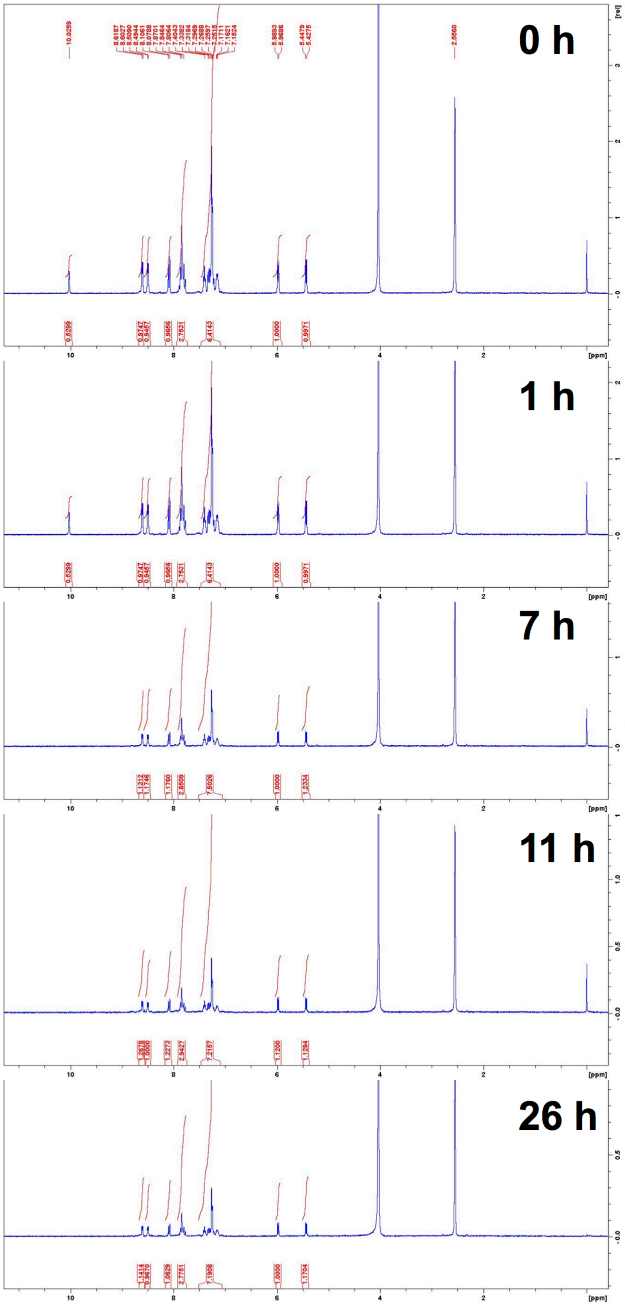

Figure S15. Stability study of PDHP in DMSO-d_6. The solution was stored in NMR tube at room temperature (21 ^oC) in DMSO/D_2O (4:1). The ^1H NMR data was collected at indicated time points. PDHP stayed to be the major component after 26 hours. **Note:** We were unable to conduct the NMR stability study of PDHP in higher percentage of water due to the compound's limited solubility. In fact, PDHP crystals were formed during the incubation. More crystals were observed with longer incubation time. This was reflected in the decrease of the ^1H NMR signal. After 26 h, the crystals were collected and redissolved in DMSO/D_2O (4:1) at 37 ^oC. Based on 1H NMR, the crystals were still PDHP.

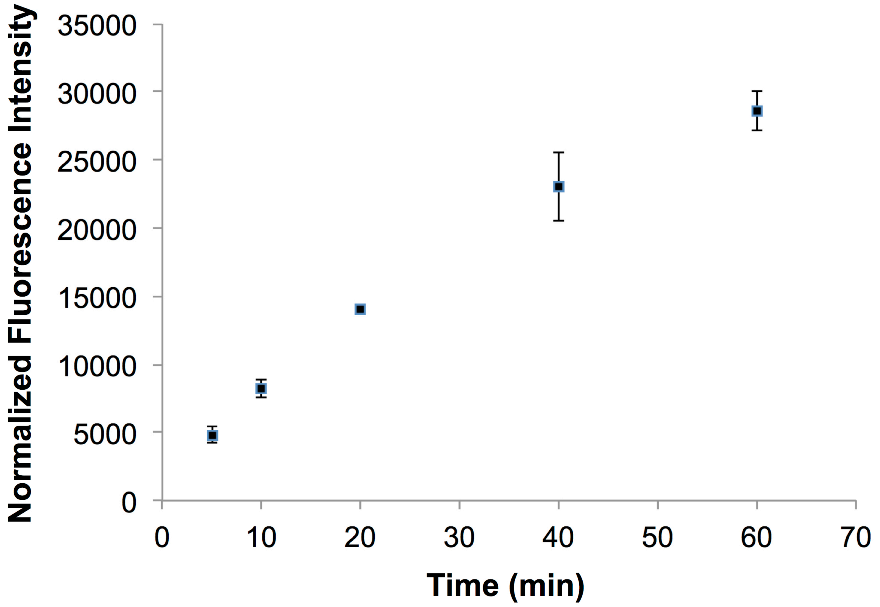

Figure 16. Reaction progress of sfGFP-N149KStyr labeling with 250 μM of Tet. Following labeling reactions, protein samples were denatured by heating, analyzed by SDS-PAGE, and imaged by GE Typhoon imager (Excitation/Emission filter: LPB (510LP)). The fluorescence intensity of each labeled protein band was measured by ImageJ. The fluorescence intensity was normalized to the amount of protein.

References

1. Gierlich, J., Burley, G. A., Gramlich, P. M. E., Hammond, D. M., and Carell, T. *Org. Lett.* **2006**, *8*, 3639-3642.
2. Niu, Z., Bruckman, M., Kotakadi, V. S., He, J., Emrick, T., Russell, T. P., Yang, L., and Wang, Q. *Chem. Commun.* **2006**, 3019-3021.
3. Finney, N. S. *Curr. Opin. Chem. Biol.* **2006**, *10*, 238-245.
4. Li, Q., Lee, J.-S., Ha, C., Park, C. B., Yang, G., Gan, W. B., and Chang, Y.-T. *Angew. Chem., Int. Ed.* **2004**, *43*, 6331-6335.
5. Thellamurege, N. M., and Li, H. *J. Chem. Phys.* **2012**, *137*, 246101.
6. Thellamurege, N. M., Si, D., Cui, F., Zhu, H., Lai, R., and Li, H. *J. Comput. Chem.* **2013**, *34*, 2816-2833.
7. (a) Schmidt, M. W., Baldridge, K. K., Boatz, J. A., Elbert, S. T., Gordon, M. S., Jensen, J. H., Koseki, S., Matsunaga, N., Nguyen, K. A., and Su, S. *J. Comput. Chem.* **1993**, *14*, 1347-1363; (b) Gordon, M. S., and Schmidt, M. W. *Theory and Applications of Computational Chemistry: the first forty years* **2005**, 1167-1189.
8. Becke, A. D. *J. Chem. Phys.* **1993**, *98*, 5648-5652.
9. Wang, N., Ju, T., Niu, W., and Guo, J. *ACS Synth. Biol.* **2014**, *4*, 207-212.
10. Liu, X., Li, J., Hu, C., Zhou, Q., Zhang, W., Hu, M., Zhou, J., and Wang, J. *Angew. Chem., Int. Ed.* **2013**, *52*, 4805-4809.
11. Zhou, Z., and Fahrni, C. J. *J. Am. Chem. Soc.* **2004**, *126*, 8862-8863.
12. Lee, Y.-J., Kurra, Y., Yang, Y., Torres-Kolbus, J., Deiters, A., and Liu, W. R. *Chem. Commun.* **2014**, *50*, 13085-13088.
13. Rieder, U., and Luedtke, N. W. *Angew. Chem., Int. Ed.* **2014**, *53*, 9168-9172.
14. Wijnen, J. W., Zavarise, S., Engberts, J. B. F. N., and Charton, M. *J. Org. Chem.* **1996**, *61*, 2001-2005.
15. Lang, K., Davis, L., Torres-Kolbus, J., Chou, C., Deiters, A., and Chin, J. W. *Nat. Chem.* **2012**, *4*, 298-304.
16. Blackman, M. L., Royzen, M., and Fox, J. M. *J. Am. Chem. Soc.* **2008**, *130*, 13518-13519.
17. Patterson, D. M., Nazarova, L. A., Xie, B., Kamber, D. N., and Prescher, J. A. *J. Am. Chem. Soc.* **2012**, *134*, 18638-18643.
18. Kamber, D. N., Nazarova, L. A., Liang, Y., Lopez, S. A., Patterson, D. M., Shih, H. W., Houk, K. N., and Prescher, J. A. *J. Am. Chem. Soc.* **2013**, *135*, 13680-13683.
19. Baskin, J. M., Prescher, J. A., Laughlin, S. T., Agard, N. J., Chang, P. V., Miller, I. A., Lo, A., Codelli, J. A., and Bertozzi, C. R. *Proc. Natl. Acad. Sci. U. S. A.* **2007**, *104*, 16793-16797.
20. Lavis, L. D., and Raines, R. T. *ACS Chem. Biol.* **2008**, *3*, 142-155.
